# Resilience and coping during protracted conflict: a comparative analysis of general and evacuees populations

**DOI:** 10.1186/s13584-024-00642-8

**Published:** 2024-10-03

**Authors:** Hadas Marciano, Shaul Kimhi, Yohanan Eshel, Bruria Adini

**Affiliations:** 1 Stress and Resilience Research Center, Tel-Hai Academic College, Kiryat Shmona, Israel; 2 The Institute of Information Processing and Decision Making, University of Haifa, Haifa, Israel; 3 ResWell Research Collaboration, Tel Aviv University, Tel Aviv, Israel; 4 Tel-Hai College, Kiryat Shmona, Israel; 5 University of Haifa, Haifa, Israel; 6 Head of the Department of Emergency Management and Disaster Management, and ResWell Research Collaboration, School of Public Health, Faculty of Medical and Health Sciences, Tel Aviv University, Tel Aviv, Israel

**Keywords:** Evacuees, General population, Distress symptoms, Hope, Societal resilience, Community resilience, Individual resilience

## Abstract

**Background:**

On October 7th, 2023, Hamas launched a surprise attack on Israel, triggering a conflict with Israel in the Gaza Strip. This ongoing war, now six months old, has also seen threats from Hezbollah in Lebanon, as well as from Yemen and Iran. The precarious security situation along Israel’s southern and northern borders led to extensive evacuations, with residents relocating within Israel under uncertain conditions concerning their return and property safety. This study compares resilience (societal, SR; community, CR; and individual, IR), hope, morale, distress symptoms (anxiety and depression symptoms), and perceived danger between general Hebrew-speaking adults and evacuee adults a few months post-conflict initiation.

**Methods:**

Data was collected using structured self-reported questionnaires focusing on resilience and coping strategies, administered through two online panel companies. The general population data was collected from January 14–21, 2024 (N = 1,360), and the evacuees’ data from March 1–9, 2024 (N = 372; 133 from the north, 239 from the south).

**Results:**

Evacuees reported lower SR and CR, hope, and morale, and higher distress symptoms and perceived danger compared to the general population. No differences in IR were found. Regression analyses identified different primary predictors of SR for each group: hope for the general population and governmental support for evacuees. Additionally, IR significantly predicted outcomes only among evacuees, whereas age, religiosity, and education were significant predictors solely in the general population. One notable similarity emerged: CR served as the second most influential predictor in both samples.

**Conclusions:**

The entire population of Israel is affected by the ongoing war, yet evacuees endure a disproportionately severe impact, with potential for increased harm as the conflict persists. The adjustment to a new wartime emergency routine is more complex for evacuees than for the general population. It is crucial for policy and decision-makers to address the distinct differences between evacuees and the general populace to effectively meet their specific needs. Yet, it should be acknowledged that the evacuees represent a heterogenic group, necessitating a detailed subdivision into subgroups to accurately assess and address their unique challenges.

## Background

On Saturday, October 7th, 2023, the Hamas (Harakat al-Muqawama al-Islamiya) movement, along with other Palestinian militant groups, initiated a surprise attack on the State of Israel. The assault commenced with the launch of thousands of rockets targeting the southern and central regions of Israel in the early morning hours. Subsequently, thousands of Hamas members, along with militants from other groups, as well as civilians, breached the border fence surrounding the Gaza Strip. The attack utilized various means of transportation, including vehicles, paragliders, boats, and on foot. Military bases and numerous settlements in the Gaza envelope, including Kibbutzs, rural villages, and cities, were intruded. Furthermore, the assault targeted the Nova Music Festival, where thousands of attendees were subjected to extreme and brutal violence. The attack resulted in a devastating loss of life, with over 1200 individuals killed, including Israeli civilians (37 of them were children), foreign nationals, and Israeli security personnel. Additionally, around 240 individuals, comprising civilians, including children, women, elderly individuals, foreign nationals, and soldiers, were taken hostage and transported to the Gaza Strip [57]. Furthermore, approximately 2000 individuals sustained injuries, including cases of sexual assault [55].

As a result of the Hamas’ surprising attack, numerous residents from the southern region of Israel were swiftly relocated to various locations across the country, often departing with nothing but the clothing on their backs. Additionally, a significant number of inhabitants from the northern region of Israel were evacuated to enable military forces to fortify the northern defense against potential incursions by Hezbollah from Lebanon. Israel is currently engaged in armed conflict with the Gaza Strip and faces aerial bombardment from Hezbollah, as well as other regions including Yemen and Iran [24]. Consequently, approximately six months following the initial attack on October 7th, 2023, a considerable number of evacuees from both the southern and northern regions of Israel remain displaced, and many of them are still residing in temporary accommodations. The duration and resolution of this forced displacement remain uncertain, leaving evacuees uncertain about when and how they may return to their homes.

The data collection of the general population sample took place on January 14–21, 2024, involving 1360 respondents. Subsequently, from March 1–9, 2024, we conducted additional data collection among 372 evacuee respondents from both the southern and northern regions of Israel, employing a similar questionnaire, facilitated by another online panel company. The current study seeks to contrast the perceptions of resilience, as well as positive and negative coping mechanisms, between the general population and the evacuees. Through the examination of these two datasets, our objective is to evaluate the hypothesis suggesting that although Israel as a whole is facing a notable security crisis, evacuees experience elevated levels of risks and instability in comparison to the general populace. Thus, we posit that they warrant particular attention from governmental bodies.

Numerous studies have explored the psychological responses of communities affected by disasters compared to neighboring communities unaffected by the events. For instance, in flood research, it was found that interpersonal resources, community social capital, and engagement were key factors for positive adaptation in affected communities, while community economic development and trust in community leadership drove adaptation in the comparison community [6], and in the context of bushfires in Australia, distress rates were consistently high among all participants, worsened by the severity of bushfire exposure [40]. Additionally, extensive literature exists on the resilience and coping strategies of evacuees displaced during various catastrophic events. Studies have focused on war evacuees [23, 42, 43, 58], evacuees from nuclear accidents [35, 44, 53], and those affected by natural disasters such as hurricanes [27, 36], earthquakes [35, 53], tsunamis [35, 53], floods [44], and volcanic eruptions [44]. However, to our knowledge, no study has directly compared coping mechanisms between the general population and evacuees during an event that impacts the entire population, though to a varied extent. This research aims to fill this gap by examining and contrasting the coping strategies employed by both groups, the general population and evacuees, in the context of such an all-encompassing event. Focusing solely on the general population may obscure the unique challenges faced by evacuees during the ongoing war. Conversely, centering solely on evacuees might exaggerate the perceived psychological impact of the conflict on the broader Israeli public. Therefore, comparing the responses of these two groups is crucial for gaining a comprehensive understanding of the situation.

### Resilience

The theoretical construct of psychological resilience has been extensively investigated in numerous studies, with various definitions proposed. One widely supported definition posits resilience as the capacity of individuals to effectively manage crises and adversities and subsequently recover to the greatest extent possible [45]. Over the years, researchers have examined different forms of resilience, including individual resilience [41], community and societal resilience [33], and organizational resilience [54], among others. In the current study, the focus lies on exploring societal, community, and individual resilience.

#### Societal resilience (SR, also referred to as ‘National resilience’)

This construct pertains to a society’s ability to navigate threats that affect either the entire society or significant portions of it. When SR is robust, society demonstrates effective adjustment and functioning in response to potentially traumatic events [33]. Canetti et al. [12] define SR as the capacity of a nation or large society to withstand hardships while maintaining its societal structural integrity (i.e., social cohesion). Over the past decade, academic discourse has seen a notable increase in interest regarding SR. Ballada et al. [7] attribute this increased interest to the rise in various crises that have threatened humanity during this period, including natural disasters such as earthquakes, volcanic eruptions, and the global COVID-19 pandemic. Additionally, crises stemming from human actions, such as major accidents, terrorist attacks, or wars, have contributed to the heightened scientific and public attention on SR. Previous research conducted during crises, such as armed conflicts or the COVID-19 pandemic, has demonstrated correlations between SR and other coping indicators. For instance, studies have found positive associations between SR and factors like hope and morale, while negative correlations were observed with distress symptoms [20, 29, 39, 56].

#### Community resilience (CR)

This construct denotes the collective capacity of a community to endure and adapt amidst stressful events or conditions, encompassing both natural disasters and human-made crises and to subsequently recover from these adversities. Eachus [18] defines CR as the ability of a community to anticipate risks, mitigate their impacts, and rapidly bounce back following such calamities. Some researchers link CR to the term “social capital” which refers to networks that link individuals through either weak or strong connections (e.g., [3, 4]). Recent research on the Ukraine-Russian conflict has indicated a positive correlation between CR and the other forms of resilience (SR and IR), as well as positive coping indicators such as hope and well-being. Conversely, CR has been found to exhibit negative associations with negative coping indicators such as psychological distress symptoms and a sense of danger [29].

In a previous study conducted during a former armed conflict, the SR and CR of Israelis residing in the Gaza envelope were compared with data previously collected from the general population. The findings revealed that while the SR level of the southern sample was significantly lower than that of the general population, the opposite trend was observed regarding CR, which was higher than that of the general population [33]. Padan and Elran [44] also reported a high CR among Gaza envelope residents during previous military clashes. In addition, Shapira [50] reported consistently relatively high CR among Gaza envelope residents across three distinct measurements, conducted during periods that varied significantly in terms of security and health threats. A similar pattern of results was observed among inhabitants of 29 communal settlements located in a regional council near the Lebanese (northern) border (Unpublished data).

#### Individual resilience (IR)

Throughout their lives, individuals often confront numerous severe incidents that they may perceive as personally traumatic events [9]. Researchers have coined the term “individual resilience” to describe the capacity to withstand and recover from such adversities. IR reflects a set of protective factors that aid individuals in adapting, ameliorating, or modifying their responses to mitigate these challenges [10]. Kimhi, Baran et al. [29] discovered positive associations between IR, SR, and CR, as well as with hope and well-being. Conversely, IR was found to exhibit negative correlations with a sense of danger and psychological distress symptoms. Direct comparisons of IR between armed conflict evacuees and the general population are undocumented. It is hypothesized that evacuees may exhibit decreased resilience, pending empirical validation.

### Coping indicators

Coping indicators can serve as markers of either positive [14] or negative [19] predictors regarding an individual’s ability to cope with a particular adversity. In the present study, we investigated two positive coping indicators, namely hope and morale, alongside two negative coping indicators, namely psychological distress symptoms and a sense of danger.

#### Hope

Hope, as defined by Snyder et al. [52], involves the anticipation that positive outcomes will manifest in the future. Conversely, Fredrickson [21] conceptualized hope as an emotional state. In our previous research endeavors, hope consistently emerged as the most robust predictor of SR [26, 39].

#### Morale

This concept initially associated with the military sphere, has been extended to encompass various domains [48]. In the context of the current study, morale is defined as a general measure of mood amidst wartime circumstances. It encapsulates the current state of mind, contrasting with hope, which pertains to perceptions regarding future prospects. Morale and hope often demonstrate a positive correlation [46].

#### Psychological distress

Psychological distress, characterized by anxiety and depression symptoms, commonly manifests in individuals facing significant threats or adversities [13]. For instance, Salari et al. [49] conducted a literature review and meta-analysis, revealing a high prevalence of anxiety and depression symptoms during the COVID-19 pandemic, with over 30% of the populations surveyed reporting such symptoms. Similarly, Levin et al. [37] demonstrated a heightened prevalence of distress symptoms across large samples in response to various disasters in different countries, including the COVID-19 pandemic in China and the UK, a Super Typhoon in the Philippines, and terror attacks in the UK, USA, and France. Distress symptoms have been found to exhibit a negative correlation with all forms of resilience, including IR, CR, and SR [34], as well as with hope [39].

#### Sense of danger

Sense of danger assesses the degree to which individuals perceive their current situation as hazardous, either for themselves or for their family members. Previous research has indicated that residents living near the southern border of Israel exhibited a heightened sense of danger compared to the general population [33]. Additionally, the sense of danger has been positively associated with psychological distress [8, 32].

## Research hypotheses

Drawing from the literature outlined above, we formulate the following hypotheses:

1. The evacuees’ sample will demonstrate lower levels of SR and IR compared to the general population. However, they are expected to exhibit higher levels of CR.
2. The evacuees’ sample will demonstrate lower levels of hope and morale compared to the general population.
3. The evacuees’ sample will exhibit higher levels of distress symptoms and sense of danger compared to the general population.
4. Consistent with previous findings from our research endeavors, we hypothesize that hope will emerge as the most salient predictor of SR within both sample groups [26, 39].

## Methods

### Participants

This study compares two samples that completed similar structured self-reported questionnaires on resilience and coping strategies during an ongoing armed conflict between Israel and Gaza (Iron Swords). The comparison between the samples was based solely on scales that were identical in both questionnaires. The data collection of the general sample data took place on January 14–21, 2024, approximately three months after the onset of the war. A total of 1,360 respondents participated in this data collection, with the questionnaire distributed through the Sekernet online panel company (https://sekernet.co.il/). The sampling utilized a stratified sampling method aligned with data from the Israeli Central Bureau of Statistics. The evacuees’ sample data was collected on March 1–9, 2024, about five months after the onset of the war, as soon as a sufficient sample of evacuees was established by the I-panel online panel company (https://www.ipanel.co.il/en/), which distributed the questionnaire to the specific evacuee respondents. A total of 372 respondents completed the questionnaire, 133 (35.8%) were evacuees from the north of Israel, and 239 (64.2%) were evacuees from the south of Israel. Eligibility criteria for participation in both samples included being an adult over 18 years old. The questionnaire received approval from the Ethics Committee of Tel Aviv University (general population: 0005985-2; evacuees: 0005985-4), and all participants provided written informed consent.

### Study tools

All the scales included in this study have been utilized in previous research and demonstrated robust reliability and validity.

#### Societal resilience

(SR; [31]). The scale comprises 16 items, e.g., “I love my country and am proud of it”. The score for each item ranges from 1 = ‘strongly disagree’ to 6 = ‘strongly agree’; a higher averaged score indicates a higher SR. The current study’s Cronbach’s alpha reliability of this tool was high in both samples (α = 0.88 and α = 0.87 for the general and evacuee samples, respectively).

#### Community resilience

(CR; [38], CCRAM). The scale consists of ten items, e.g., “I trust the decision makers in my community”, each is rated by a 5-point scale ranging from 1 = ‘strongly disagree’ to 5 = ‘strongly agree’; a higher average score indicates a higher CR. The Cronbach’s alpha reliability of this measurement in the current study was high in both samples (α = 0.93 and α = 0.94 for the general and evacuee samples, respectively).

#### Individual resilience

(IR; [1, 11, 15]). This tool is the 10-item Connor-Davidson scale (CD-RISC 10). It addresses individual feelings of ability and power in the face of difficulties, e.g., “I manage to adapt to the changes”. The responses ranged from 1 = ‘not true at all’ to 5 = ‘generally true’; a higher mean score indicates a higher IR. The Cronbach’s alpha reliability in the current study was high in both samples (α = 0.92 and α = 0.89 for the general and evacuee samples, respectively).

#### Hope

Based on previous scales [25]. The scale includes five items, e.g., “I will emerge strengthened from the current crisis”. The scale ranged from 1 = ‘very little hope’ to 5 = ‘very much hope’, thus a higher mean score indicates a higher level of hope. The Cronbach’s alpha reliability of this measurement was high in both samples (α = 0.94 and α = 0.92 for the general and evacuee samples, respectively).

#### Psychological distress symptoms

This scale includes items concerning anxiety and depression symptoms (BSI, [17]). It presents eight items describing different symptoms (four for anxiety symptoms, e.g. “In the past days, how much were you distressed by nervousness”, and four for depressive symptoms, e.g. “In the past days, how much were you distressed by feeling lonely”). This scale ranges from 1 = ‘not at all’ to 5 = ‘to a very large extent’, thus a higher average score indicates higher distress levels. The Cronbach’s alpha reliability of this measurement was high in both samples (α = 0.93 and α = 0.91 for the general and evacuees samples, respectively).

#### Sense of danger

[51]. This tool includes seven items, e.g. “To what extent do you feel that your life is in danger?”. The scale ranges from 1 = ‘not at all’ to 5 = ‘to a very large extent’. A higher average score indicates higher levels of sense of danger. The Cronbach’s alpha reliability of this measurement was good in both samples (α = 0.85 for both samples).

#### Morale

This variable was gathered via a single item asking respondents about their current morale level, with responses ranging from 1 = ‘very poor’ to 5 = ‘very good’.

#### Demographic details

The following demographic details were gathered from both samples: age, gender, religiosity, support of the current government, education level, and a question regarding being negatively affected during the war. This question was phrased as: “Have you or any of your first-degree relatives been negatively affected during the war?” with a “yes” or “no” response option. For those who answered “yes,” a follow-up question asked respondents to select any relevant options from the following: physically injured, mentally injured, property damage, or other (with an open response). The distribution of the demographic characteristics of each sample is presented in Table 1.

**Table 1:** Demographic characteristics of the general sample (N = 1360) and the evacuees’ sample (N = 372)

Variable	Group	General sample (N = 1360) Number (%)	Evacuees’ sample (N = 372) Number (%)	T-test or Chi-square comparisons
Age	18–30	295 (21.7)	121 (32.5)	
	31–40	272 (20.0)	105 (28.2)	
	41–50	284 (20.9)	71 (19.1)	
	51–60	229 (16.8)	46 (12.4)	
	61–85	280 (20.6)	29 (7.8)	
	Mean (S.D)	45.39 (15.76)	39.06 (13.48)	t = 7.08*
Gender	1. Men	731 (53.8)	121 (32.5)	χ^2^ = 52.64*
	2. Women	629 (46.2)	251 (67.5)	
Degree of religiosity	1. Secular	642 (47.2)	191 (51.3)	
	2. Traditional	426 (31.3)	97 (26.1)	
	3. Religious	181 (13.3)	61 (16.4)	
	4. Very religious	111 (8.2)	23 (6.2)	
	Mean (S.D)	1.82 (.95)	1.77 (.94)	NS
Support of the government	1. Greatly oppose	396 (29.1)	113 (30.4)	
	2. Oppose	212 (15.6)	63 (16.9)	
	3. Intermediate	330 (24.3)	89 (23.9)	
	4. Support	235 (19.3)	78 (21.0)	
	5. Greatly support	159 (11.7)	29 (7.8)	
	Mean (S.D)	2.69 (1.37)	2.59 (1.32)	NS
Education level	1. Elementary school	22 (1.6)	2 (0.5)	
	2. High school	278 (20.4)	93 (25.0)	
	3. Partial academic	360 (26.5)	90 (24.2)	
	4. Bachelor’s degree	447 (32.9)	132 (35.5)	
	5. ≥ Master’s degree	253 (18.6)	55 (14.8)	
	Mean (S.D)	3.46 (1.06)	3.39 (1.03)	NS
Been affected by the war	1. Yes	68 (5.0)	277 (74.5)	χ^2^ = 883.55*
	2. No	1292 (95.0)	95 (25.5)	

*p < 0.0001

## Results

### Demographic differences

One notable demographic difference between the two samples is the gender distribution. The general sample comprised slightly more men than women, while the evacuees’ sample consisted of significantly more women than men (see Table 1); a chi-square test showed that this difference was significant (χ^2^ = 52.64, *p* < 0.0001). Furthermore, another crucial difference between the two samples, which is particularly pertinent to the current findings concerns the extent to which individuals were affected by the war. While only 5% of the general sample responded affirmatively, a striking percentage of 74.5% of the evacuees’ sample answered “yes”. A chi-square test revealed that this disparity was statistically significant (χ^2^ = 883.55, *p* < 0.0001). Moreover, t-test indicated a significant difference in the mean age of the samples. The evacuees’ sample was significantly younger than the general sample (t = 52.64, *p* < 0.0001, Cohen’s d = 0.414).

### Resilience and coping differences

Seven separate t-tests were conducted to compare the means of the two samples on the seven different variables. Except for IR, all variables exhibited significant differences with small or medium effect sizes between the samples (see Table 2). Specifically, the SR and CR of the evacuees were lower than those of the general population. Similarly, the levels of hope and morale among the evacuees were also lower than those of the other sample. In addition, the levels of distress symptoms and the sense of danger were higher among the evacuees compared to the general population.

**Table 2:** Resilience and coping differences between the two samples

Measurement	Variable	General sample (N = 1360)		Evacuees’ sample(N = 372)		t	Cohen’s d
		M	SD	M	SD		
Resilience	Individual	3.57	.72	3.52	.71	1.7	.068
	Societal	3.86	.81	3.49	.83	7.7**	.452
	Community	3.50	.82	3.32	.96	3.7**	.216
Positive coping	Hope	3.68	.90	3.56	.10	2.1*	.126
	Morale	3.29	.90	3.18	.98	2.1*	.124
Negative coping	Distress	2.33	.95	2.82	.97	8.8**	.515
	Sense of Danger	2.64	.85	2.94	.91	6.0**	.353

**p < .0001

*p < 0.05

### Predicting societal resilience

Two separate linear regression analyses were conducted to assess the predictability of SR in each of the samples, incorporating all other variables. Initially, all demographics and psychological coping variables were included, followed by the inclusion of only variables that were significant in either of these analyses. The final regression data for each sample, presented from the highest to the lowest predicting variable, for each sample, are displayed in Table 3.

**Table 3:** Results of multiple linear regression analyses for predicting SR in each sample separately

	Predicting Variable	β	% Explained variance
General sample (N = 1360)	Hope	.383***	R^2^ = .438
	Community resilience	.265***	
	Government support	.233***	
	Age	.148***	
	Religiosity	− .099***	
	Education	− .070**	
	Sense of danger	− .063**	
	Individual resilience	− .025 (NS)	
Evacuees’ sample (N = 372)	Government support	.313***	R^2^ = .465
	Community resilience	.295***	
	Sense of danger	− .192***	
	Hope	.151**	
	Individual resilience	.092*	
	Education	− .044 (NS)	
	Age	.039 (NS)	
	Religiosity	− .021 (NS)	

***p < .0001

**p < 0.01

*p < 0.05

In the general population sample, the best predictor was the level of hope, with higher levels of hope associated with greater SR. Conversely, in the evacuees’ sample, the best predictor was the level of support for the Israeli government, indicating that higher support for the government was associated with higher SR. Notably, hope ranked as the fourth predictor in the evacuees’ sample, contrasting with its primary predictive role in the general sample. Additionally, in both samples, CR emerged as the second most significant predictor, with higher CR linked to greater SR. Three demographic variables significantly predicted SR in the general sample: lower education levels, lower religiosity levels, and older age were associated with higher SR. However, these variables did not significantly predict SR in the evacuees’ sample. In contrast, IR was not significant in the general sample but was in the sample of the evacuees, indicating higher IR associated with greater SR. Comparable explained variances were found in both analyses: 43.8% for the general sample and 46.5% for evacuees.

## Discussion

The study compared data from two samples during the ongoing conflict war between Israel and the Hamas movement in Gaza. One represented the general Hebrew-speaking population of Israel, while the other comprised individuals evacuated from southern and northern regions, enduring displacement five months post-conflict onset. Both samples completed comparable questionnaires via distinct online panel providers. Both measurements were conducted during the implementation of a new wartime emergency routine in the country. The data for the general population were collected about three months after the surprise attack by Hamas on Israel, and the data for the evacuees were collected two months later.

Following the first hypothesis, the sample of evacuees exhibited significantly reduced levels of SR in comparison to the general population. This observation corroborates prior research, notably the findings indicating reduced SR among inhabitants from both the southern and northern regions of Israel [33], Unpublished data). However, diverging from the initial hypothesis, evacuees also displayed lower levels of CR compared to the general population, while their IR did not exhibit a significant disparity. The lower CR levels may be attributable to the disintegrating of evacuee communities from their original configuration. While some groups relocated collectively, others dispersed across various regions of the country. Although many evacuees maintain affiliations with their former community leaders or authorities, the communal cohesion of cohabitation has been dissolved. Additionally, the previously observed high resilience in communities along Israel’s northern and southern borders may be attributed to their perceived success in confronting repeated security challenges. This sustained exposure likely fostered a sense of empowerment, which in turn enhanced their CR [44]. This insight is reinforced by interviews conducted in 2020 with adolescents and their parents living in the Gaza envelope, who consistently emphasized the importance of material and social support systems available to them during periods of escalation [2]. However, the October 7th attack and its aftermath have unfortunately made it difficult to continue viewing the coping of these communities as “successful”. This experience may have negatively impacted the perceived CR of many evacuees. The observed parity in IR between evacuees and the general population, contrary to expectations of lower IR among evacuees, may stem from the collective trauma resulting from Hamas’s attack on October 7th, 2023. This impact appears consistent across both directly and indirectly affected individuals [28]. Additionally, the initial higher IR in evacuee communities, such as those near the Lebanese border who have previously shown higher resilience compare with the general population despite ongoing conflicts (Unpublished data), suggests that residents of high-risk areas may have inherently higher IR. This pattern suggests that the apparent parity might reflect a relative decline in evacuee resilience from their initially higher levels. Confirming and understanding these dynamics require further longitudinal research with consistent participants.

In alignment with the second hypothesis, the sample of evacuees demonstrated significantly reduced levels of hope and morale compared to the general population. Similarly, congruent with the third hypothesis, evacuees exhibited significantly higher levels of distress symptoms and a higher sense of danger compared to the general population sample. These findings were expected given the manifold challenges encountered by evacuees relative to the broader population. A substantial majority of the evacuees, exceeding 74%, reported direct personal impacts from the conflict. Some experienced the loss of family members, bore witness to harrowing scenes, or sought refuge in secure spaces within their residences while Hamas members infiltrated their communities or even their homes, committing violence, destruction, and hostage-taking. Moreover, all evacuees underwent displacement from their homes, leaving behind their residences amidst the uncertainty of the safety of their properties in bombarded areas, compelled to embark on a new life in unfamiliar places with uncertain timelines [5].

Contrary to the fourth hypothesis, hope emerged as the most significant predictor of SR solely within the general population, with no such association observed within the sample of evacuees. Instead, the level of support for the Israeli government emerged as the most influential variable predicting SR among evacuees, indicating that higher government support correlated with higher SR. This finding diverges from previous research wherein hope consistently emerged as the primary predictor of SR across various studies [26, 39]. For instance, hope has been consistently identified as the foremost predictor of SR in studies examining diverse crises and geographical contexts [16, 22, 30]. Thus, the current revelation regarding the evacuees, wherein hope ranked as the fourth predictor of SR and government support superseded it as the primary predictor, represents a notable deviation from established patterns. It is reasonable to infer that evacuees perceive a significant reliance on the decisions and actions undertaken by the government. This dependency stems from their need to procure essential support while displaced and to facilitate their eventual return to their residences. The effectiveness of the Israeli government’s decisions and actions is of utmost importance in this context. Hence, individuals trusting the Israeli government tend to have higher SR, while distrust correlates with decreased SR.

Furthermore, this pattern may result from certain individuals’ firsthand experiences, perceiving state institutions, including the military and security forces, as inadequately providing timely assistance in critical moments on October 7th 2023. This profound realization may have led them to be unwilling to restore their trust in state institutions, regardless of their level of hope for a better future. To examine this proposition, the aforementioned regression analysis was conducted separately for individuals from the southern region, who were indeed affected by the violence of Hamas, and for those from the northern region, who, despite being evacuated from their homes, did not experience direct acts of violence at the onset of the war. The findings of the regression analysis align with the suggested explanation. The most significant predictor of SR is government support in both groups (β = 0.311, *p* = 0.0001 and β = 0.312, *p* = 0.0001, for the north and south evacuees, respectively). However, hope emerged as the second most influential predictor of SR in the northern region sample (β = 0.249, *p* = 0.003), whereas this relationship was not significant in the southern region sample (β = 0.083, *p* = 0.206). These results suggest that evacuees from the north and the south should not be considered as a homogeneous subpopulation. Further studies involving larger samples of evacuees from both regions could provide additional evidence to confirm this conclusion.

Nevertheless, it is noteworthy to mention that the second most influential predictor of SR in both samples was CR, with higher CR linked to increased SR. This finding is in line with previous findings regarding the association between CR and SR (e.g., [47]. This insight is significant and encouraging, as CR is a variable that can be addressed through straightforward measures. For instance, the government can allocate funds to local authorities to bolster CR or to provide aid for their inhabitants, and, even more importantly, efforts should prioritize maintaining community cohesion during evacuations. Furthermore, while IR emerged as a predictor for SR solely in the evacuees’ sample, demographic variables such as age, religiosity, and education, which were significant predictors of SR in the general population, did not show significant associations with SR in the evacuees’ sample. The difference in the significance of age may stem from the notable age gap between the samples, with evacuees being significantly younger than the general population. However, the other discrepancies between the samples cannot be solely attributed to differences in sample characteristics. Therefore, it is reasonable to suggest that during periods of adversity, such as those faced by evacuees, demographic variables may not exert a substantial influence on preserving SR.

### Limitations

Several limitations of the present study warrant acknowledgment: (a) While the study utilized an online panel survey designed to capture diverse segments of Israeli society or the evacuee subpopulation, it cannot be definitively concluded that the samples are fully representative. For instance, it is evident that the “very religious” sub-population is underrepresented (at least in the general population sample, where it should comprise approximately 20%), as well as the Arab population. Additionally, the new evacuee-specific panel was relatively small, and in an effort to maximize data collection, no formal sampling considerations were applied. As a result, the sample is not fully representative and differs from the general population sample in terms of age and gender. It remains unclear how these potential biases may affect the findings and to what extent, but it may lead to an underestimation of the negative impacts of evacuation, given that these populations may be differently affected due to various socio-economic and cultural factors. To address this, we recommend that future studies focus on more specific sub-populations and employ alternative sampling methods to improve representativeness. (b) As with any correlational study, no direct cause-and-effect relationships can be inferred from the current study. (c) Although the study sheds light on the importance of investigating resilience within the specific subgroup of evacuees, several considerations should be kept in mind. Firstly, the data from the evacuee sample was collected approximately two months after the collection from the general population sample. Ideally, both data sets would have been gathered during the same period; however, due to logistical constraints, this was not feasible. It should thus be considered that the evacuee sample was exposed to war and displacement for a longer time compared to the general population. Secondly, the evacuee population is heterogeneous, with potentially significant differences depending on their regions of residence (north versus south). Thirdly, there are additional subgroups likely to be profoundly affected by the conflict, yet they are not comprehensively represented in our current samples. These include individuals who were injured or have relatives affected by the events of October 7th, 2023, as well as those impacted by subsequent developments in the conflict. Furthermore, attention should be directed towards individuals actively involved in combat or with family members serving as soldiers. Moreover, the current study’s samples consisted solely of Hebrew-speaking adults, but it is imperative to also examine children and adolescents, as well as Arabic-speaking Israelis, including subgroups of Arabic speakers, such as Bedouins and Druzes. Future studies should focus on these subgroups specifically to provide a more nuanced understanding of the psychological repercussions of the war.

## Conclusions

Based on the current findings, several conclusions can be drawn: (1) While the entire population of Israel is affected by the war, evacuees from both southern and northern regions experience a notably heightened impact. (2) It is reasonable to assume that the longer the evacuation persists, the greater the harm will become. (3) While the general population seems to demonstrate strong sympathy and solidarity with the evacuees, the implementation of a new wartime emergency routine in Israel enables the general population to maintain relatively higher levels of societal and community resilience compared to the evacuees. This is accompanied by lower levels of distress symptoms and perceptions of danger among the general population. It can be inferred that the living situation of evacuees complicates their adjustment to this new wartime emergency routine, contrasting with the general population’s adaptation. (4) It is apparent that evacuees themselves constitute a heterogeneous group, warranting careful subdivision into subgroups. While a primary division based on original residence (northern or southern regions) is evident, further divisions should be considered, such as age groups and family status. (5) Ultimately, additional studies are necessary to assess the situation of other special groups, including those directly affected by the initial events of October 7th, 2023 and subsequent developments of the war, children and adolescents, Israeli Arabs, etc.

Policy and decision-makers should be attuned to the variability that distinguishes evacuees from the general populace, considering their unique and heightened needs when designing appropriate response strategies. This consideration is vital not only for supporting vulnerable groups in the present but also for preparing them for potential challenges that may arise in the near future.

## Data Availability

The datasets used and/or analyzed during the current study are available from the corresponding author on reasonable request.

## Abbreviations

BSI: Brief symptom inventory
CD-RISK: Connor-Davidson resilience scale
CR: Community Resilience
HAMAS: Harakat al-Muqawama al-Islamiya
IR: Individual Resilience
SR: Societal Resilience
